# Novel Pharmaceuticals in Appetite Regulation: Exploring emerging gut peptides and their pharmacological prospects

**DOI:** 10.1002/prp2.1243

**Published:** 2024-07-17

**Authors:** Igor Rubinić, Marija Kurtov, Robert Likić

**Affiliations:** ^1^ Department of Basic and Clinical Pharmacology and Toxicology, Faculty of Medicine University of Rijeka Rijeka Croatia; ^2^ Clinical Pharmacology unit Clinical Hospital Center Rijeka Rijeka Croatia; ^3^ Division of Clinical Pharmacology and Toxicology, Department of Internal Medicine University Hospital “Sveti Duh” Zagreb Croatia; ^4^ Department of Internal Medicine School of Medicine University of Zagreb Zagreb Croatia

**Keywords:** amylin, anti‐obesity pharmaceuticals, cholecystokinin, ghrelin, gut peptides, peptide YY

## Abstract

Obesity, a global health challenge, necessitates innovative approaches for effective management. Targeting gut peptides in the development of anti‐obesity pharmaceuticals has already demonstrated significant efficacy. Ghrelin, peptide YY (PYY), cholecystokinin (CCK), and amylin are crucial in appetite regulation offering promising targets for pharmacological interventions in obesity treatment using both peptide‐based and small molecule‐based pharmaceuticals. Ghrelin, a sole orexigenic gut peptide, has a potential for anti‐obesity therapies through various approaches, including endogenous ghrelin neutralization, ghrelin receptor antagonists, ghrelin O‐acyltransferase, and functional inhibitors. Anorexigenic gut peptides, peptide YY, cholecystokinin, and amylin, have exhibited appetite‐reducing effects in animal models and humans. Overcoming substantial obstacles is imperative for translating these findings into clinically effective pharmaceuticals. Peptide YY and cholecystokinin analogues, characterized by prolonged half‐life and resistance to proteolytic enzymes, present viable options. Positive allosteric modulators emerge as a novel approach for modulating the cholecystokinin pathway. Amylin is currently the most promising, with both amylin analogues and dual amylin and calcitonin receptor agonists (DACRAs) progressing to advanced stages of clinical trials. Despite persistent challenges, innovative pharmaceutical strategies provide a glimpse into the future of anti‐obesity therapies.

## Graphical abstract

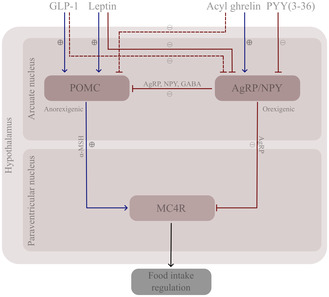

**Abbreviations**
AgRP: agouti‐related peptide
AMY1‐3: amylin receptors 1‐3
AP: area postrema
BMI: body mass index
CART: cocaine‐ and amphetamine‐related transcript
CCK: cholecystokinin
CGRP: calcitonin gene‐related peptide
CNS: central nervous system
CRH: corticotrophin‐releasing hormone
CT: calcitonin
CTR: calcitonin receptor
DA: dopamine
DACRA: dual amylin and calcitonin receptor agonists
DIO: diet‐induced obese
DPP‐4: dipeptidyl peptidase‐4
EMA: European Medicines Agency
FDA: Food and Drug Administration
GABA: gamma‐aminobutyric acid
GHSR: growth hormone secretagogue receptor
GHSR1a: growth hormone secretagogue receptor
GIP: gastric inhibitory polypeptide
GIPR: gastric inhibitory polypeptide receptor
GLP‐1: glucagon‐like peptide‐1
GLP‐1R: glucagon‐like peptide‐1 receptor
GOAT: ghrelin O‐acyltransferase
KBP: KeyBiosciencePeptide
LEAP2: liver‐expressed antimicrobial peptide 2
LPBN: lateral parabrachial nucleus
MC4R: melanocortin‐4 receptor
NE: norepinephrine
NEP: neutral endopeptidase
NPY: neuropeptide Y
NTS: nucleus tractus solitarius
PAM: positive allosteric modulators
POMC: proopiomelanocortin
PYY: peptide YY
RAMP1‐3: receptor activity‐modifying proteins 1‐3
sCT: salmon calcitonin
SPM: Spiegelmers
α‐MSH: α‐melanocyte‐stimulating hormone

## 1 INTRODUCTION

Obesity, a multifactorial health condition characterized by an abnormal or excessive accumulation of body fat, poses a considerable health risk.^1^ There is a relentless increasing trend of obesity,^2^ with obesity prevalence expected to exceed 20% by 2025 in many European countries.^3^ This obesity epidemic is contributing to multiple noncommunicable diseases, including diabetes, cardiovascular, respiratory, gastrointestinal, and musculoskeletal issues, psychiatric conditions, as well as various types of cancer.^4^ In 2019 alone, it contributed to an estimated 5.0 million obesity‐related deaths globally.^5^ Besides detrimental health effects, it is negatively affecting society's economic landscape.^6^, ^7^, ^8^

The challenge of obesity is rooted in a complex interplay of both modifiable and non‐modifiable factors, including genetic predisposition, sedentary lifestyles, excessive calorie consumption, inadequate sleep, pharmaceuticals, medical conditions, socioeconomic disparities, ethnic backgrounds, psychosocial stressors, endocrine‐disrupting chemicals, and the composition of the gastrointestinal microbiome, among others.^9^, ^10^ Human appetite regulation is governed by the complicated coordination between the central nervous system (CNS) and peripheral hormones. Any imbalances in this system can result in increased food intake, exceeding the body's energy expenditure, and ultimately lead to weight gain.^11^

Nomenclature of Targets and Ligands: Key protein targets and ligands in this article are hyperlinked to corresponding entries in http://www.guidetopharmacology.org, the common portal for data from the IUPHAR/BPS Guide to PHARMACOLOGY (Harding et al., 2018), and are permanently archived in the Concise Guide to PHARMACOLOGY 2019/20 (Alexander et al., 2019)^12^, ^13^

### 1.1 Appetite regulation mechanisms

Obesity is a result of a prolonged energy intake and expenditure imbalance.^14^ While our evolutionary advantage once lay in storing a modest amount of fat to endure periods of famine, the control mechanisms limiting fat accumulation appear distorted due to our societal and developmental progress, largely eliminating the threat of predation.^15^

Several factors, including environmental cues, physiological signals, psychological nuances, and socio‐cultural influences, collectively form a web of inputs that our CNS processes in regulating feeding behavior and, consequently, body weight.^16^, ^17^

Food regulation depends on the interplay between the CNS, gastrointestinal system, and endocrine system. Within this complex network, gut peptides represent messengers that harmonize the inputs important for food intake, primarily via centers located in the hypothalamus and brainstem.^18^ Their functions are mediated mainly by modulating the production of neuropeptides, proteins synthesized by neurons exhibiting synaptic, paracrine, and (neuro)endocrine functions, such as agouti‐related peptide (AgRP), neuropeptide Y (NPY), cocaine‐ and amphetamine‐related transcript (CART), and proopiomelanocortin (POMC).^19^, ^20^ Of note, several other neuropeptides with important roles in maintaining energy homeostasis have been identified, offering promising avenues for the development of novel anti‐obesity drugs. This potential is exemplified by setmelanotide, a melanocortin‐4 receptor (MC4R) agonist approved for syndromic obesity. However, further research is needed to fully exploit the therapeutic potential of neuropeptide modulation in combating obesity.^20^, ^21^

Gut peptides have been discussed as a potential pharmacological target since the 1960s when the “gut–brain axis” was beginning to unravel.^22^ The importance of gut peptides in regulating weight can be illustrated by changes after bariatric surgery. Even though other significant factors include reduced absorption surface, differences in bile acids, and gut microbiota, changes in levels of gut peptides seem crucial for weight regulation.^23^ It was shown that newer methods of gastric operations can avoid nutrient malabsorption but still result in significant weight loss due to the changes in the gut peptide secretions.^24^

### 1.2 Current obesity pharmacotherapy

Several drugs have been approved by the Food and Drug Administration (FDA) and the European Medicines Agency (EMA) as anti‐obesity pharmaceuticals. The timeline of drug approval, their mechanisms of action, and indications are outlined in Table 1. Additionally, metreleptin, a leptin analogue, and the previously mentioned setmelanotide, an MC4R agonist, are approved for syndromic obesity.^25^

**TABLE 1:** Current FDA‐ or EMA‐approved non‐syndromic anti‐obesity medications.

Drug	Approval		Mechanism of action	Indications		Ref.
	FDA	EMA		FDA	EMA	
Orlistat	1999	1998	Inhibitor of gastrointestinal lipases	Obesity management including weight loss and weight maintenance when used in conjunction with a reduced‐calorie diet Indicated to reduce the risk for weight regain after prior weight loss	In conjunction with a mildly hypocaloric diet for the treatment of obese patients (BMI ≥30 kg/m^2^), or overweight patients (BMI > 28 kg/m^2^) with associated risk factors	[47, 48]
Phentermine/Topiramate	2012	Not approved^a^	CNS stimulant, NE agonist/GABA agonist, glutamate antagonist	An adjunct to a reduced‐calorie diet and increased physical activity for chronic weight management in adults with an initial BMI of: ≥30 kg/m^2^ (obese) or >27 kg/m^2^ (overweight) in the presence of at least one weight‐related comorbidity^b^	/	[49, 50]
Naltrexone/Bupropion	2014	2015	mu‐opioid receptor antagonist/neuronal DA and NE reuptake inhibitor	An adjunct to a reduced‐calorie diet and increased physical activity for chronic weight management in adults with an initial BMI of: ≥30 kg/m^2^ (obese) or >27 kg/m^2^ (overweight) in the presence of at least one weight‐related comorbidity^b^		[51, 52]
Liraglutide	2014	2015	GLP‐1 analogue	An adjunct to a reduced‐calorie diet and increased physical activity for chronic weight management in adult patients with an initial (BMI) of ≥30 kg/m^2^ (obese) or >27 kg/m^2^ (overweight) in the presence of at least one weight‐related comorbidity^b^ An adjunct to a healthy nutrition and increased physical activity for weight management in adolescent patients from the age of 12 years and above with: obesity (BMI corresponding to ≥30 kg/m^2^ for adults by international cut‐off points) and body weight above 60 kg^c^		[53, 54, 55]
Semaglutide	2021	2021	GLP‐1 analogue			[56, 57]
Tirzepatide	2023	Not approved^d^	GIP and GLP‐1 receptor agonist	An adjunct to a reduced‐calorie diet and increased physical activity for chronic weight management in adults with an initial body mass index (BMI) of: ≥30 kg/m^2^ (obese) or >27 kg/m^2^ (overweight) in the presence of at least one weight‐related comorbidity^b^	/	[58]

Abbreviations: BMI, body mass index; CNS, central nervous system; DA, dopamine; EMA, European Medicines Agency; FDA, Food and Drug Administration; GABA, gamma‐aminobutyric acid; GIP, gastric inhibitory polypeptide; NE, norepinephrine.

^a^ EMA refused authorization in 2012 and 2013 due to concerns about psychiatric and cardiovascular effects.^50^

^b^ Weight‐related comorbidities are specifically listed for distinct drugs and differ for FDA‐ and EMA‐issued approval. They can include hypertension, dyslipidemia, type 2 diabetes mellitus, prediabetes, obstructive sleep apnea, and/or cardiovascular disease.

^c^ Not in FDA approval for semaglutide.

^d^ It was approved in 2022 for type 2 diabetes by both EMA and FDA. EMA has issued an opinion on a change to this medicine's authorization on November 09, 2023 to include weight management.^59^

In the context of gut peptides, current anti‐obesity medications primarily involve drugs that target the incretin hormones glucagon‐like peptide‐1 (GLP‐1) and gastric inhibitory polypeptide (GIP).^25^, ^26^

GLP‐1, formed through posttranslational modifications of the proglucagon molecule,^27^ is expressed in pancreatic α‐ and intestinal L‐cells, as well as in the brainstem.^28^ The primary source of circulating GLP‐1 are gut epithelial cells,^29^ whereas within the CNS, it is predominantly found in the nucleus tractus solitarius, an area crucial for energy homeostasis.^30^ Its secretion is stimulated by the presence of nutrients in the digestive system.^29^ Upon binding to its receptor, GLP‐1R, a G protein‐coupled receptor, important metabolic functions, including stimulating insulin secretion, inhibiting glucagon synthesis, reducing food intake, delaying gastric emptying, and promoting pancreatic β‐cell proliferation are initiated.^27^ Its effect on food intake involves a combination of anorexic effects in the CNS, and gastrointestinal effects, such as delayed gastric emptying.^30^ However, the therapeutic utility of the natural GLP‐1 molecule is limited by its short half‐life due to rapid degradation by dipeptidyl peptidase‐4 (DPP‐4) and possibly neutral endopeptidase (NEP), as well as rapid renal clearance.^27^ Various strategies are employed to extend the half‐life of GLP‐1 for pharmaceutical use. GLP‐1 agonists already used in obesity treatment, semaglutide and liraglutide, are engineered with fatty‐acid acylation to prevent degradation by oligomer forming, with semaglutide additionally undergoing N‐terminal modification to further inhibit DPP‐4 proteolysis.^27^ Both molecules show a significant effect in achieving weight loss, simultaneously improving glycemic control, and reducing cardiovascular risk.^31^, ^32^, ^33^, ^34^, ^35^

GIP is primarily synthesized in duodenal and jejunal K‐cells through posttranslational modification of its precursor molecule, proGIP, with fat ingestion serving as a key stimulus for its secretion.^27^, ^36^ Similar to GLP‐1, GIP undergoes degradation by DPP‐4 and rapid renal clearance, precluding its use as a pharmaceutical agent.^37^, ^38^ Acting via the GIP receptor (GIPR), GIP mediates various functions which include stimulation of insulin and glucagon secretion, and regulation of lipid and energy metabolism.^27^ Initially considered an obesogenic hormone due to its role in promoting fat deposition and elevated secretion in obese individuals, GIPR antagonism was initially explored.^39^, ^40^ However, long‐term GIP agonism has been shown not to promote food intake or adiposity; instead, it leads to a negative energy balance, especially when combined with GLP‐1 agonists, resulting in significant weight loss, possibly through GIPR desensitization.^40^, ^41^, ^42^ Tirzepatide, a GIPR/GLP‐1R agonist, has demonstrated efficacy in reducing body weight in both murine models and humans.^27^, ^43^, ^44^, ^45^, ^46^

This review will shift its focus toward exploring alternative gut peptides with the potential to influence appetite and food intake offering effective avenues for treating obesity.

## 2 GHRELIN

### 2.1 Ghrelin physiology

Ghrelin, a ligand for the growth hormone secretagogue receptor (GHSR or GHSR1a), was identified in 1999 while its impact on metabolism and obesity was established in 2000.^60^, ^61^ By 2001, studies on human subjects had demonstrated ghrelin's role in stimulating appetite and increasing food intake, and lower levels of ghrelin were found in obese individuals.^62^, ^63^

Comprising 28 amino acids, ghrelin undergoes post‐translational modification through acylation, specifically at its third serine residue. Acylation, catalyzed by the enzyme ghrelin O‐acyltransferase (GOAT), is crucial for the hormone's binding to its receptors and subsequent downstream signaling.^64^, ^65^ Unexpectedly, des‐acyl ghrelin, the non‐acylated form of ghrelin with distinct physiological actions, is found in larger quantities.^66^, ^67^

The primary source of ghrelin production is the gastric fundus, where it is secreted by PD‐1 cells with additional expression found in the small intestine, pancreas, testes, and kidney.^66^, ^68^, ^69^

#### 2.1.1 Ghrelin's role in stimulating appetite

**FIGURE 1:**
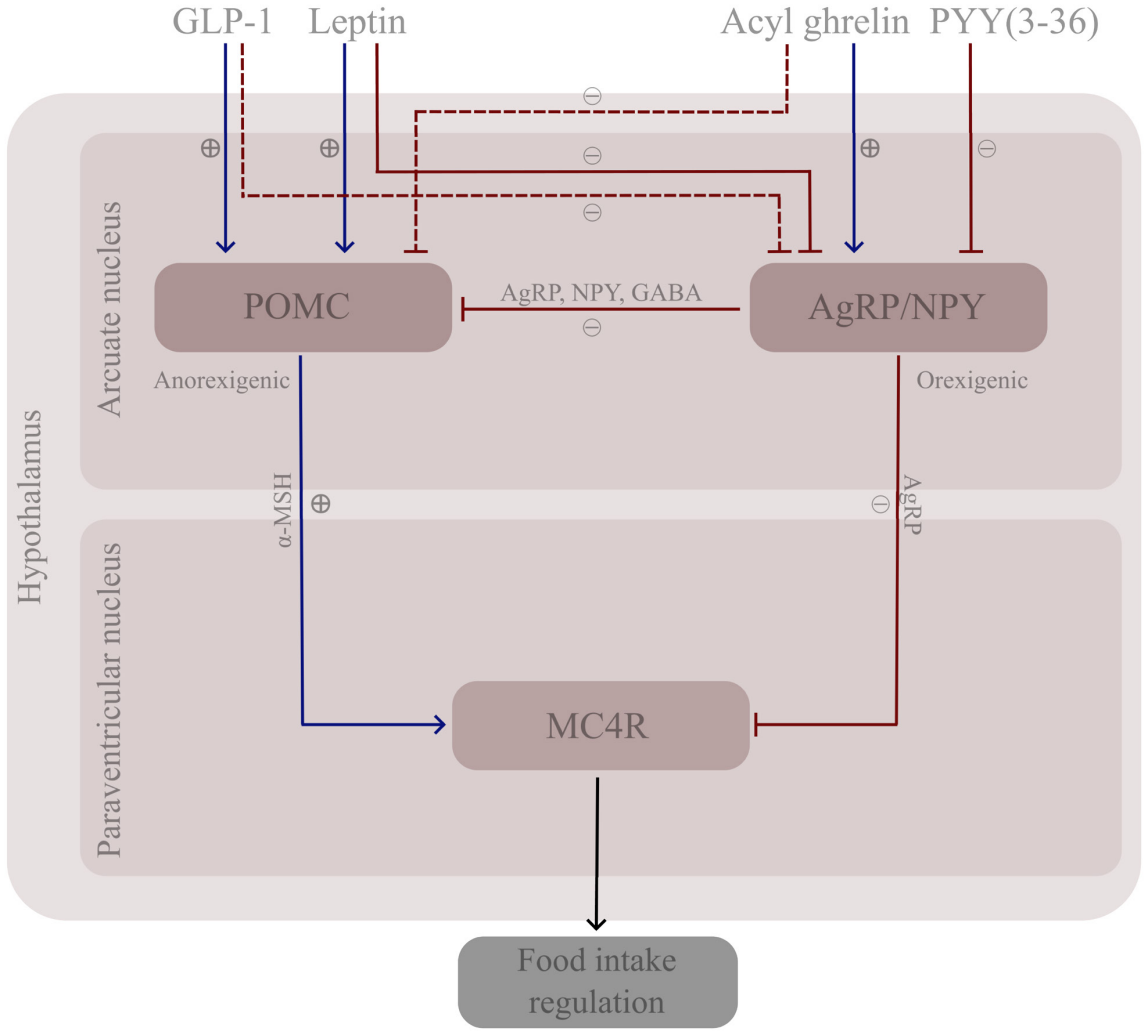
Hormonal regulation of the hypothalamic melanocortin system. The function of MC4R is influenced by orexigenic AgRP/NPY neurons, which inhibit it, and anorexigenic POMC neurons, which stimulate it. Gut peptides, such as ghrelin, PYY, and GLP‐1, as well as other factors, for example, leptin, modulate AgRP/NPY and POMC activity impacting food intake. AgRP/NPY are stimulated by ghrelin and inhibited by PYY, GLP‐1 (indirectly), and leptin. POMC are stimulated by GLP‐1 and leptin, while ghrelin indirectly inhibits it.^77^, ^78^, ^79^ There are conflicting reports on PYY's direct effect on POMC,^80^, ^81^ while the precise effects of amylin are still under investigation.^82^ AgRP, agouti‐related peptide; GABA, gamma‐aminobutyric acid; GLP‐1, glucagon‐like peptide‐1; MC4R, melanocortin‐4 receptor; NPY, neuropeptide Y; POMC, proopiomelanocortin; PYY, peptide YY; α‐MSH, α‐melanocyte‐stimulating hormone.

Ghrelin's nickname, the “hunger hormone,” reflects its crucial role in stimulating appetite. Upon release, ghrelin traverses the bloodstream to reach the hypothalamus, a brain region crucial for appetite control. There, it stimulates NPY and AgRP neurons in the arcuate nucleus.^70^, ^71^ NPY/AgRP neurons release NPY, AgRP, and gamma‐aminobutyric acid (GABA) which have inhibitory effects on POMC. This inhibitory action prevents the release of α‐melanocyte‐stimulating hormone (α‐MSH) from POMC, hindering its binding to the MC4R and disrupting the generation of anorexigenic signal.^72^, ^73^ Additionally, AgRP is an inverse agonist of α‐MSH, blocking its action on the MC4R.^73^ The process is illustrated in Figure 1. Acyl ghrelin also antagonizes opposing signals from anorexigenic molecules such as CART, leptin, corticotrophin‐releasing hormone (CRH), and others.^67^ Additionally, ghrelin engages brain regions associated with reward, intensifying the desire for calorically dense and palatable foods.^74^, ^75^ Des‐acyl ghrelin, contrastingly, exerts opposing effects – decreases food intake, fat mass, and gastric emptying.^76^

Dual influence on the hypothalamus and reward centers creates a potent drive for increased food intake, contributing to the persistence of obesity‐related challenges.

#### 2.1.2 Regulation of ghrelin levels and factors influencing its release

Ghrelin secretion increases during fasting, peaks before meals, and declines postprandially.^83^, ^84^, ^85^ There are many factors impacting the secretion of ghrelin. It is stimulated by fasting, beta‐adrenergic and muscarinic stimulation, and by hormones such as cholecystokinin, glucagon, or estrogen. Inhibition is mediated by alpha‐adrenergic effects, glycemia, insulin, long‐chain fatty acids, leptin, and somatostatin.^66^ The order in which macronutrients are ingested also affects ghrelin levels, as well as external influences such as sleep patterns^86^, ^87^ and stress.^88^, ^89^ Reduced concentrations of acyl ghrelin and des‐acyl ghrelin typically indicate substantial energy reserves. However, obese individuals exhibit lower baseline levels of acyl ghrelin, experiencing postprandial declines similar to those observed in lean individuals but of shorter duration, possibly due to the inhibitory effect of increased insulin levels.^63^, ^90^ Moreover, the potential roles of growth hormone and leptin as regulators of ghrelin levels were suggested; however, the studies did not conclusively establish their involvement.^91^

### 2.2 Potential pharmacological interventions targeting ghrelin for appetite control

Modulating the ghrelin pathway through different drug functions (agonist, inverse agonist, antagonist) as well as the target itself can have different impacts. The potential therapeutic applications of drugs affecting the ghrelin pathway extend beyond obesity to a variety of disorders including anorexia, gastrointestinal issues, inflammation, substance abuse, cardiovascular, pulmonary, and renal diseases, as well as neurological disorders such as epilepsy, Alzheimer's disease, multiple sclerosis, and Parkinson's disease.^92^, ^93^, ^94^, ^95^ While GHSR agonists, such as ibutamoren ^96^ or anamorelin,^97^ are already on the market, due to inconsistent data on safety and effectiveness, there are yet no anti‐obesity drugs targeting the ghrelin signaling cascade.^98^, ^99^, ^100^ Besides therapeutics, macimorelin, a GHSR agonist developed by Aeterna Zentaris, is utilized for diagnosing growth hormone deficiency.^92^, ^101^

#### 2.2.1 Neutralization of circulating ghrelin

Among the first in the field were drugs designed to neutralize the circulating ghrelin. Initial attempts focused on passive immunization through transferring anti‐ghrelin antibodies, leading to the inhibition of feeding in animal models.^102^ Subsequently, the investigation of ghrelin vaccines, designed to elicit an immune response to ghrelin, proved effective in rodents and pigs, resulting in reduced food intake, diminished hypothalamic orexigenic signals, and increased energy expenditure.^103^, ^104^, ^105^, ^106^ In humans, a Phase I/IIa trial with CYT009‐GhrQb, developed by Cytos Biotechnology, was conducted in 2006, involving 87 obese patients, but the trial was discontinued as no additional weight loss was observed compared to the control group.^106^, ^107^ Another approach to inactivating endogenous ghrelin involved the use of RNA Spiegelmers (SPM), single‐stranded mirror image oligonucleotides that bind to ghrelin, rendering it inactive.^108^ A representative of this mechanism is NOX‐B11‐3 which demonstrates activity in rodents with elevated ghrelin levels during food restriction (dieting). However, it failed to influence basal food intake in non‐food‐deprived rats.^109^, ^110^

#### 2.2.2 Ghrelin receptor antagonists

GHSR antagonists have demonstrated promising outcomes in enhancing glucose tolerance, suppressing appetite, and fostering weight loss in preclinical models.^110^ Several non‐peptide small molecules, including JMV2959, AZ‐GHS‐38, JMV 3002, and others were under investigation for obesity in the preclinical development phases.^92^, ^111^ Current investigations are focused on peptide‐based molecules such as liver‐expressed antimicrobial peptide 2 (LEAP2), initially described by their antimicrobial properties, but recently recognized as an inverse GHSR agonist and a reversible ghrelin antagonist.^112^ Given that LEAP2 opposes ghrelin, there is optimism that increasing the LEAP‐2/ghrelin ratio could be an effective approach to combat obesity.^113^ While studies have shown that LEAP2 lowered postprandial plasma glucose and reduced food intake in 20 healthy men without changing the ghrelin levels, further research, as well as drug optimization due to short half‐life are necessary.^99^, ^114^

#### 2.2.3 Ghrelin O‐acyltransferase (GOAT) inhibitors

An alternative strategy for addressing obesity involves manipulating the ghrelin pathway by targeting GOAT, thereby reducing the levels of acyl ghrelin, the active form that binds to the ghrelin receptor. Theoretically, the continual production of des‐acyl ghrelin, which cannot activate the GHSR, is anticipated, while the production of acyl ghrelin would cease. Consequently, a reduction in appetite, the promotion of negative energy balance, and an enhancement of insulin sensitivity and release should be expected.^66^ Various classes of GOAT inhibitors, such as peptide and peptidomimetic, terpenoid and steroid‐based, and small molecule inhibitors, have already been developed. Expectedly, these inhibitors should exhibit minimal side effects, given that ghrelin is the sole substrate for GOAT in humans.^115^ Several human trials targeting GOAT have been recently performed or are still ongoing for various indications.^116^, ^117^, ^118^ The latest research, featuring Boehringer Ingelheim's BI 1356225, demonstrated a remarkable reduction (>80%) in the ratio of acyl ghrelin to des‐acyl ghrelin.^119^ However, there was no observed impact on body weight, hunger/satiety, control of eating, or overall energy intake during the studied 4‐week period. It was suggested that an increase in des‐acyl ghrelin rather than a decrease in acyl ghrelin could lead to weight loss.^119^ This suggests that GOAT might not be a significant therapeutic target for obesity treatment.

#### 2.2.4 Functional inhibitors

As previously mentioned, des‐acyl ghrelin serves as a functional inhibitor of acyl ghrelin.^100^ Notably, the des‐acyl ghrelin analogue livoletide (AZP‐531), developed by Millendo Therapeutics, demonstrated successful treatment of hyperphagia in patients with Prader–Willi syndrome, resulting in a significant reduction in body weight.^100^ However, the subsequent phase 2b/3 ZEPHYR trial did not yield a statistically significant improvement in hyperphagia and food‐related behaviors, leading to the discontinuation of further development.^120^, ^121^

### 2.3 Challenges in targeting ghrelin for obesity therapy

Developing drugs targeting ghrelin for obesity treatment presents a formidable challenge due to the intricate regulation of this hormone,^122^ the contrasting functions of acyl ghrelin and des‐acyl ghrelin in appetite and fat storage,^123^ the redundancy in appetite control systems,^124^ and significant individual variability, particularly evident in obese versus lean individuals^90^ among other factors. Additionally, safety concerns stem from the multiple physiological effects of ghrelin^125^ and the absence of standardized tests to measure the concentration of acylated ghrelin^126^ adds a layer of complexity to the development process.

## 3 PEPTIDE YY

### 3.1 Structure and production of peptide YY

Peptide YY (PYY), also known as peptide tyrosine tyrosine, is a 36‐amino acid hormone within the NPY family.^127^, ^128^ Predominantly secreted by enteroendocrine cells, particularly L‐cells in the distal gut, PYY is also produced in smaller quantities within the CNS and by α‐, PP‐, and δ‐cells in the pancreas.^129^ PYY manifests two main isoforms – PYY(1‐36), and the biologically active PYY(3‐36) that regulates appetite and satiety. The conversion of PYY(1‐36) to PYY(3‐36) is facilitated by DPP‐4 through the removal of the N‐terminal Try1‐Pro2 dipeptide.^130^, ^131^ PYY therefore shares a synthesis location with GLP‐1 and undergoes degradation by the same enzyme.^129^ Modest or negligible weight loss observed with DPP‐4 inhibitors, despite heightened incretin levels, may, at least in part, be attributable to reduced levels of anorectic PYY(3‐36).^132^, ^133^

### 3.2 Peptide YY's physiology

The release of both PYY isoforms is tied to nutrient intake, with proteins and calorie content being the most potent stimulators of secretion peaking approximately 90 minutes after a meal.^134^, ^135^, ^136^ Individuals with obesity exhibit lower fasting PYY(3‐36) levels and a reduced peak response, requiring double caloric intake to achieve levels equivalent to lean individuals.^137^

Upon release into the bloodstream, PYY(3‐36) exerts its effects by binding to the G‐protein‐coupled Y receptors,^129^, ^138^ exerting anorexigenic effects through the Y2 receptor in arcuate nucleus, and possibly the activation of inhibitory neurons in cortex, subcortical regions, and the brainstem^139^, ^140^ for which PYY(3‐36) shows high affinity.^140^ In the arcuate nucleus, PYY(3‐36) silences NPY/AgRP neurons, indirectly activating POMC neurons, as shown in Figure 1.^26^ These complex interactions suppress orexigenic signals, resulting in diminished feelings of hunger and an enhanced sense of fullness, a phenomenon demonstrated through direct administration of PYY(3‐36) in rodents, primates, and humans.^130^, ^135^, ^141^ Peripheral administration of PYY(1‐36) in rodents shows a less pronounced anorectic effect.^141^ By binding to Y receptors, PYY also exerts influence on gastric motility and secretion, contributing to the deceleration of the digestive process and prolonging the sensation of satiety.^129^, ^142^ Furthermore, through the Y1/2 receptor, PYY assumes a role in safeguarding pancreatic beta cells by preventing apoptosis, thereby preserving beta‐cell mass—an essential feature in preventing or slowing the progression of diabetes.^143^, ^144^

### 3.3 Exploring pharmacological strategies to enhance PYY's appetite‐suppressing effects

#### 3.3.1 PYY(3‐36) administration

Expectedly, initial studies exploring the potential of PYY as an anti‐obesity drug focused on peripheral administration of PYY(3‐36), which effectively reduces weight gain by inhibiting food intake in rodents.^135^ The same was observed in humans, including obese individuals, suggesting a potential link between PYY deficiency and the development of obesity.^145^ Intravenous infusion of PYY(3‐36) discovered a dose‐dependent reduction in energy intake that persisted for 24 hours but was associated with gastrointestinal side effects.^146^ Subsequently, subcutaneous administration was found ineffective even though an increase in PYY plasma levels was detected, possibly due to degradation or biological inactivation.^146^, ^147^ Intranasal application of PYY(3‐36) was evaluated in a study involving 12 obese subjects over 12 weeks, with two different dosage regimens tested: 200 μg three times daily and 600 μg three times daily.^148^ Although a relevant increase in plasma PYY was noted for both dosing schedules, the lower dose failed to yield significant weight loss, while the higher dose was poorly tolerated due to nausea and vomiting. In summary, the clinical utility of PYY(3‐36) administration faces obstacles due to its short biological half‐life and gastrointestinal side effects such as nausea, vomiting, and abdominal discomfort.^129^ Therefore, the main hope for targeting the PYY system is the development of PYY(3‐36) analogues.^26^, ^149^

#### 3.3.2 PYY(3‐36) analogues

PYY analogues can be made by several approaches resulting in proteolytic stability and improved selectivity.^150^, ^151^, ^152^ Selective Y2 receptor PYY analogues have already shown significant potential in reducing body weight in diet‐induced obese (DIO) rodents, especially when combined with semaglutide.^153^, ^154^ A long‐acting conjugate comprising a cyclized PYY(3‐36) analogue and a functionally silent monoclonal antibody, strategically added to enhance half‐life and decrease subcutaneous absorption rates, has demonstrated the ability to reduce food intake without heightening the risk of emesis in rhesus macaques.^155^

A synthetic Novo Nordisk's PYY analogue, PYY1875/NNC0165‐1875 (NN9775‐4708), was examined in combination with semaglutide for obesity treatment but was recently discontinued following the completion of Phase II trials.^156^, ^157^ Several other PYY analogues are in the earlier studies of development.^131^

### 3.4 Challenges in targeting peptide YY for obesity therapy

PYY's limited half‐life, susceptibility to enzymatic degradation, and propensity to induce gastrointestinal side effects hinder its direct administration. A possibility is the development of stable PYY(3‐36) analogues, which aim to enhance proteolytic stability, prolong half‐life, and reduce side effects, therefore overcoming the limitations associated with the hormone's natural form. Similar to other gut peptides, the physiological differences between animal models and humans present a significant challenge, complicating the replication of preclinical results with equivalent efficacy in human trials.

## 4 CHOLECYSTOKININ

### 4.1 Structure and production of cholecystokinin

Cholecystokinin (CCK) is a peptide hormone with diverse roles in digestion and appetite regulation.^158^ Synthesized as a larger precursor molecule, pre‐pro‐CCK transforms into proCCK by removing the signal sequence.^159^ Further modifications, including endoproteolytic activity, are crucial in the creation of distinct active forms of CCK.^160^ Multiple molecular forms of CCK exist, categorized by the number of amino‐acid residues in the final peptide, ranging from 4 to 83. The predominant molecular form is CCK‐58, with CCK‐8 and CCK‐33 being less prevalent, alongside several other identified variants. Gastrin, due to structural similarity, exhibits weak CCK‐like activity, and vice versa.^161^

CCK is primarily synthesized in the I‐cells of the duodenum and jejunum. These cells are primarily stimulated by the lipid and protein content of a meal. However, due to the presence of distinct surface receptors in various parts of the small intestine, different nutrients may also trigger the release of CCK.^162^, ^163^, ^164^, ^165^ In addition, CCK is also synthesized in various other tissues, including the adrenal glands, thyroid gland, pituitary gland, central and peripheral nervous systems, urogenital tract, cardiovascular system, and immune system, indicating a wide array of physiological functions.^163^

### 4.2 Cholecystokinin's physiology

CCK's digestive functions are integral to nutrient absorption. Via the CCK1 receptor, also termed CCK‐A (alimentary) receptor, it stimulates the gallbladder to release bile, promoting the digestion and absorption of lipids. Moreover, via the same receptor, it prompts the pancreas to secrete digestive enzymes, delays gastric emptying, as well as gastric acid secretion.^163^ The CCK1 receptor is also expressed in the vagal afferents, brainstem, and hypothalamus which is thought crucial for appetite suppression.^77^ Stimulation of the CCK1 receptor activates vagal afferent neurons, triggering an upregulation in the synthesis of CART, an anorexigenic neuropeptide promoting appetite suppression in the CNS.^166^

CCK2 receptors, also referred to as CCK‐B (brain) receptors or gastrin receptors, represent the main CCK receptor in the brain.^167^ Consequently, these receptors are associated with neurotransmission, anxiety regulation, dopamine activity, GABA release, and nociception modulation.^163^, ^168^, ^169^ The same receptor is present in the pancreatic islet cells.^163^ CCK influences insulin secretion significantly, as elevated CCK levels have been shown to stimulate insulin release, while the absence of CCK results in a reduction in pancreatic islet size and beta cell mass.^170^

In 1973, CCK emerged as the pioneering gut peptide demonstrating the ability to inhibit food intake, a groundbreaking finding observed through intraperitoneal CCK administration in rats.^171^ This appetite‐suppressing effect has since been observed in various animal models and human studies.^168^ As CCK's satiety‐inducing effects are mediated through visceral afferent nerves, transmitting signals to the CNS, eliminating the need to traverse the blood–brain barrier, and simplifying drug development.^162^ Stimulation of the CCK1 receptor is crucial for an anti‐obesity effect, while it is simultaneously essential to avoid activation of the CCK2 receptor, as its agonists may induce anxiety and panic.^172^ Importantly, CCK1 stimulation without simultaneous CCK2 stimulation is feasible due to different ligand recognition properties.^162^

### 4.3 Investigating potential pharmacological interventions targeting cholecystokinin for appetite regulation

#### 4.3.1 Cholecystokinin analogues and small CCK1 receptor agonists

Due to its influence on appetite regulation, structurally modified and enzyme‐resistant versions of CCK,^168^, ^173^, ^174^, ^175^ as well as small molecule CCK1 receptor agonists^176^, ^177^, ^178^ have been developed and demonstrated efficacy as appetite suppressors in animal models. In human trials, the CCK1 receptor agonist and CCK2 receptor antagonist, 1,5‐benzodiazepine (GI181771X by GSK), underwent a phase II trial involving 701 patients but exhibited no significant effect on body weight. Of note, two cases of gallstone‐related pancreatitis were reported.^179^ Considering the limited efficacy comparable to acute dieting, substantial side effects, and the potential for tumorigenesis associated with CCK1 receptor agonists, recent literature proposes an exploration into the development of safer and more effective alternatives.^162^

#### 4.3.2 Positive allosteric modulators

Biased agonists or positive allosteric modulators (PAM) emerge as potential candidates, some of them already being investigated.^162^, ^180^, ^181^ PAMs lack inherent CCK receptor agonistic properties; instead, they function as amplifiers of endogenous CCK signaling, offering temporal control and a concise duration of action during physiologically relevant periods.^181^

We have not identified any ongoing human trials investigating the impact of CCK as a potential treatment for obesity.

### 4.4 Challenges in targeting CCK pathway for anti‐obesity therapy

CCK presents a complex scenario for therapeutic development, given its existence in multiple molecular forms, synthesis across various tissues, and involvement in diverse physiological functions, from the gastrointestinal tract to the CNS. These complicated characteristics pose a formidable challenge in designing effective therapeutics. Safety concerns further compound the issue, with CCK1 receptor agonists potentially inducing gastrointestinal side effects and being linked to carcinogenic effects. Additionally, CCK2 receptor agonists are associated with anxiety, raising concerns, especially when contemplating chronic use. Once again, the translation of successful outcomes from animal models to human trials remains a significant hurdle, impeding progress. Still, the emergence of potentially safer alternatives, such as PAMs, may provide a way of utilizing CCK's effects.

## 5 AMYLIN

### 5.1 Amylin's structure, production, and physiology

Amylin, or islet amyloid polypeptide, is a 37‐amino acid peptide that is co‐secreted with insulin in response to ingested nutrients.^182^ It is primarily synthesized in pancreatic β‐cells and, to a lesser extent, in other tissues, originating from an 89‐amino acid prohormone. This prohormone undergoes several modifications to form the active hormone.^183^ Following secretion, it has various roles, including slowing gastric emptying, suppressing glucagon secretion, and initiating an anorectic signal, all essential for maintaining glucose homeostasis.^184^

Amylin receptors are composed of the calcitonin receptor (CTR), a G protein‐coupled receptor, and one of three receptor activity‐modifying proteins (RAMP1‐3) that amplify the binding of amylin to CTR. Together, they form three distinct types of amylin receptors (AMY1‐3). Different AMY receptors have different affinity for amylin, as well as other agonists such as calcitonin, calcitonin gene‐related peptide (CGRP), and adrenomedullin.^183^, ^185^, ^186^, ^187^, ^188^

Amylin's impact on satiety is regulated by receptors in the CNS. Peripheral amylin, binding to receptors in the area postrema (AP), transmits signals through the nucleus tractus solitarius (NTS) and lateral parabrachial nucleus (LPBN) to forebrain regions, such as the central amygdala, thereby influencing eating behavior and metabolic pathways, possibly by modulating the hedonic aspects of eating.^184^, ^186^, ^189^ The impact on POMC and NPY neurons in the arcuate nucleus is not yet completely clear.^82^ Amylin's effect in reducing the intake of food is rapid and dose dependent.^190^ It is not clear if inhibiting gastric emptying is a result of central, vagal, or local factors, yet it additionally promotes satiety and delays the entry of nutrients into the intestine, dampening the glucose peak. Moreover, amylin inhibits glucagon secretion further impacting the pathophysiology of diabetes.^186^, ^190^, ^191^ It is established that amylin does not directly affect α‐cells, and a possible explanation includes its impact on the hindbrain and subsequent effects on the vagus nerve, yet the mechanism of amylin's glucagenostatic effect is not yet clear.^183^ Amylin exhibits a synergistic effect when combined with leptin, PYY, CCK, GLP‐1, and other anorexigenic molecules.^183^

### 5.2 Exploring pharmacological strategies to utilize amylin for appetite control and glucose regulation

Various animal models have demonstrated that the administration of amylin leads to the suppression of feeding and induces weight loss.^192^, ^193^, ^194^ The main limitation of human amylin is its short half‐life, a result of renal clearance and proteolysis,^195^ as well as its propensity to aggregate into fibrils that have no therapeutic value and are even harmful.^196^

#### 5.2.1 Amylin analogues

By altering the amylin molecule, amylin analogues with extended half‐lives and non‐aggregating properties present a potentially viable obesity treatment. The first and yet only approved amylin analogue is pramlintide, created by modifying three amino acids, with potency similar to human amylin, but significantly reduced aggregation potential.^183^, ^190^, ^197^ Utilized alongside insulin it improves glycemic control by reducing appetite and glucagon secretion while slowing gastric emptying and potentially providing protection to endothelial cells.^190^, ^198^ The main limitations involve a brief half‐life, necessitating administration with each meal, and potential gastrointestinal side effects, most commonly nausea, yet anorexia and vomiting are also reported.^199^ Moreover, the perceived risk of severe hypoglycemia could be a factor hindering approval in countries beyond the United States.^200^, ^201^ While it exhibits promise in weight reduction, newer drugs with extended half‐lives, resulting in improved adherence, could be more appropriate.^185^, ^202^

Various amylin modifications have been explored to extend the duration of amylin to make it suitable for chronic use. An illustrative example is davalintide, an amylin receptor agonist created by merging amylin with salmon calcitonin, exhibiting increased potency and a prolonged half‐life. In animal models, it has demonstrated a reduction in food intake lasting up to 23 hours and a more significant weight loss. However, the development of the drug was discontinued due to its inability to showcase superiority over pramlintide.^203^

Several other amylin analogues, including ZP4982 and ZP5461 developed by Zealand Pharma, as well as BI 473494, a collaborative effort between Zealand Pharma and Boehringer Ingelheim have been investigated. ZP4982 and ZP5461 demonstrated effective glycemic control and induced weight loss in preclinical models, ZP4982 even being superior to liraglutide,^185^, ^204^ yet it was recently discontinued.^205^ BI 473494 progressed to Phase I, involving 16 healthy participants, with one participant developing a serious side effect, acute polyneuropathy.^206^ Further drug development was discontinued, with Boehringer Ingelheim pursuing other obesity drugs, while the global rights to the amylin analogue program remained with Zealand Pharma.^207^, ^208^, ^209^

Petrelintide (ZP8396), another amylin analogue developed by Zealand Pharma, boasts a 10‐day half‐life attributed to acetylation, rendering it suitable for convenient once‐weekly administration.^210^ Demonstrating efficacy in reducing body weight and enhancing glucose homeostasis in animal models, petrelintide recently underwent assessment in a Phase I study involving 56 subjects. The results indicated a dose‐dependent weight loss effect with mild adverse events.^211^ Phase 2 is anticipated to start in 2024.^210^

#### 5.2.2 Dual amylin and calcitonin receptor agonists

A promising strategy in addressing obesity by targeting the amylin pathway involves utilizing dual amylin and calcitonin receptor agonists (DACRAs), that show superior metabolic effects compared to amylin analogues.^212^, ^213^ DACRAs effects were first noticed in salmon calcitonin (sCT) which elicits metabolic effects by engaging both calcitonin (CT) and amylin receptors, in contrast to rat CT, which lacks affinity for amylin receptors.^190^

The initial DACRAs, known as KBPs (for KeyBiosciencePeptide), exhibited promising outcomes in animal models.^214^ Prolonged action analogues, denoted as KBP‐A, were derived through acetylation, facilitating convenient once‐weekly administration.^214^ Preclinical studies show the efficacy of several KBPs, such as KBP‐066,^215^ KBP‐066A,^216^ KBP‐088,^217^ and KBP‐336.^218^ No clinical trials investigating obesity with KBPs were identified; however, clinical trials for type 2 diabetes are currently assessing the efficacy of KBP‐042 and KBP‐089. In a Phase I study involving 37 healthy subjects, KBP‐042 exhibited a favorable safety profile at tested doses, albeit higher doses were associated with nausea and vomiting.^219^ However, Phase II trials for KBP‐042 in type 2 diabetes did not reveal a significant improvement in HbA1C levels.^219^, ^220^ Eli Lilly discontinued the development of KBP‐042 in 2019, opting instead for KBP‐089 which showed better results.^203^ Even though KBP‐089 had good results in rodent models, the Phase I study was terminated due to strategic reasons attributed to limited pharmacodynamic effect.^221^

The most promising drug currently under investigation for obesity targeting the amylin pathway is cagrilintide (formerly AM‐833), developed by Novo Nordisk, demonstrating effectiveness in preclinical studies, particularly when combined with semaglutide.^222^ A Phase II trial revealed its significant impact on reducing body weight while maintaining a favorable tolerability profile.^223^ Another Phase 2 study demonstrated the considerable effects of the cagrilintide/semaglutide combination on both body weight and HbA1c level, despite HbA1c differences comparable to semaglutide alone.^224^ Currently, multiple Phase III studies involving cagrilintide/semaglutide are underway,^225^, ^226^, ^227^, ^228^ including plans for a head‐to‐head study with tirzepatide, a recently approved anti‐obesity drug by Eli Lilly.^229^, ^230^

### 5.3 Challenges in targeting the amylin pathway for anti‐obesity treatment

Several challenges emerge when the amylin pathway is considered as a target for anti‐obesity treatment. Human amylin poses issues due to its short half‐life, necessitating frequent administration, which, in turn, affects patient adherence. Furthermore, its inclination to aggregate into harmful fibrils is another obstacle. Adverse effects, including gastrointestinal side effects, the risk of hypoglycemia with pramlintide, along with a serious side effect reported in a trial involving an amylin analogue, are examples of additional hurdles that may impede regulatory approval. Moreover, in common with other gut peptides, there are many examples of successful animal models, but it seems difficult to translate the results to human clinical trials. However, emerging alternatives, such as DACRAs and synergistic combinations with medications targeting other gut peptide pathways, hold promise as potential solutions to the outlined limitations.

## 6 CONCLUSION

Obesity, a global health concern associated with numerous complications, demands innovative solutions to address its rising prevalence. Ghrelin, PYY, CCK, and amylin have promising prospects for novel pharmaceutical interventions in appetite regulation and obesity treatment. Ongoing research and innovative approaches, including ghrelin receptor modulators, stable PYY analogues, positive allosteric modulators for CCK, and advanced amylin analogues or DACRAs, showcase the potential for more effective and targeted anti‐obesity medications. The evolving landscape of pharmaceutical development offers hope for overcoming existing challenges and improving outcomes in the fight against obesity.

## AUTHOR CONTRIBUTIONS

All authors contributed substantially to the conception of the work. IR and MK wrote the first draft of the work. RL performed critical revision. All authors gave final approval of the version to be published.

## FUNDING INFORMATION

This research received no specific grant from any funding agency in the public, commercial, or not‐for‐profit sectors.

## CONFLICT OF INTEREST STATEMENT

None to declare.

## ETHICS STATEMENT

Not applicable.

## PATIENT CONSENT STATEMENT

Not applicable.

## PERMISSION TO REPRODUCE MATERIAL FROM OTHER SOURCES

Not applicable, all materials are from publicly available sources.

## CLINICAL TRIAL REGISTRATION

Not applicable.

## Data Availability

Data sharing does not apply to this article as no new data were created or analyzed in this study.
